# Three-Dimensional Printed Customized Scaffolds Covered with Decellularized Bone Extracellular Matrix for Open-Wedge High-Tibial Osteotomy

**DOI:** 10.3390/bioengineering11111129

**Published:** 2024-11-08

**Authors:** Geunseon Ahn, Jun-Young Kim, Jin-Hyung Shim, Sang-Hyun An, Junsik Kim, Changhwan Kim, In-Gyu Lee, Jung-Min Shin, Byunghoon Lee

**Affiliations:** 1 Research Institute, T&R Biofab Co., Ltd., Seongnam-si 13486, Republic of Korea; gsahn84@gmail.com (G.A.); greatfrog22@gmail.com (C.K.); iglee@tnrbiofab.com (I.-G.L.); 2 Department of Orthopaedic Surgery, School of Medicine, Catholic University of Daegu, Daegu 42472, Republic of Korea; dr.junyoung@gmail.com; 3 Department of Mechanical Engineering, Tech University of Korea, Siheung-si 15073, Republic of Korea; happyshim@tukorea.ac.kr; 4 Preclinical Research Center, Daegu Gyeongbuk Medical Innovation Foundation (K-MEDI Hub), Daegu 41061, Republic of Korea; ash4235@kmedihub.re.kr (S.-H.A.); skim@kmedihub.re.kr (J.K.); 5 Department of Rheumatology, Nowon Eulji Medical Center, Eulji University, Seoul 34824, Republic of Korea; 6 Department of Orthopaedic Surgery, Gil Medical Center, Gachon University College of Medicine, Incheon 21936, Republic of Korea

**Keywords:** three-dimensional printing, scaffold, high-tibial osteotomy, decellularized bone extracellular matrix

## Abstract

Void fillers are required for osseous gaps generated after orthopedic procedures as medial open-wedge high-tibial osteotomy (MOWHTO) to provide sufficient structural support and a rapid osteosynthesis. We developed a novel three-dimensional (3D) printing-based platform technology using the customized 3D scaffolds covered with polycaprolactone (PCL)/β-tri-calcium phosphates (β-TCP)/bone decellularized extracellular matrix (dECM) for use as bone substitute scaffold, which can be effectively exploited to estimate the calculated correction angle with preoperative simulations. PCL/β-TCP/bone dECM scaffolds demonstrated significantly higher cell contain levels in cell seeding efficiency, excellent proliferation capacity, and promotion of early osteogenic differentiation compared with PCL/β-TCP scaffolds. The scaffolds promoted bone mineralization at the early time points of an in vivo study (8 weeks) and exhibited biodegradable properties (38% for 16 weeks). The correction angle measured after osteotomy using 3D printed scaffolds was estimated with high accuracy with low errors (10.3° ± 0.9°) and was not significantly different even in the presence of lateral cortical hinge fractures. The customized 3D scaffold enriched with PCL/β-TCP/bone dECM yielded excellent cell seeding efficiency, proliferation capacity, early osteogenic differentiation, and bone mineralization outcomes. It is expected to solve the disadvantages related to bone union in MOWHTO and to replace autografts in the future.

## 1. Introduction

High-tibial osteotomy (HTO) has emerged as a valid and reliable proxy for the alleviation of pain, improvement of function, and delay of joint degeneration in patients with osteoarthritis (OA). Notably, it decreases the magnitude of external knee adduction moments during walking, thereby reducing the dynamic load on the medial tibiofemoral joint [1,2,3].

At first, the lateral closed wedge technique was introduced. However, several disadvantages, such as fibula osteotomy, lateral muscle detachment, or dissection of the peroneal nerve, were indicated. Recently, the number of medial open wedge high-tibial osteotomies (MOWHTOs) has increased with the use of the locking plate Tomofix^®^. However, several concerns exist owing to the documented losses of correction and delayed union or nonunion of the open wedge gap after osteotomy. Therefore, there is considerable interest in the use of bone substitutes for the improvement of bone repair.

Various void fillers can be used, including autograft, allograft, bone substitute fillers, and noncalcium–phosphate-based void fillers. Nicholas and Omer [4,5] reported in their systematic review that autografts exhibit superior union results compared with allografts. The use of autografts yielded more favorable outcomes than synthetic bone substitutes with regard to the rates of delayed union or nonunion and mean time to union. Therefore, it has been indicated that there are no definitive advantages for OWHTO with any bone void filler in terms of union rates. Additionally, given the loss of correction and the use of synthetic bone substitutes, OWHTO cannot be recommended.

However, autografts were indicated as the treatment choice for salvage procedures in cases of nonunion following OWHTO owing to the harvest-site morbidity [6,7,8]. The use of bone void filler for large osteotomy gaps was also recommended [9]. Meanwhile, the addition of tri-calcium phosphate (TCP) into osteotomy gaps as a bone substitute filler does interfere with normal bone healing [10]. Therefore, the use of bone substitute fillers in conjunction with advanced technologies has been proposed for the substitution of autografts.

Three-dimensional (3D) printing technologies have been used to fabricate the tissue engineered scaffolds because of the fabrication possibility of the 3D free-form structures with fully interconnected pores for adequate tissue regeneration [11,12]. Owing to the inherent brittleness of ceramic-based scaffolds in load-bearing scenarios [13,14], like HTO regions, in this study, we fabricated a customized 3D scaffold with the synthetic polymer polycaprolactone (PCL) and the ceramic beta-tricalcium phosphate (β-TCP) for HTO via 3D printing technology.

Generally, tissues in the body consist of tissue-specific extracellular matrix (ECM) that can modulate cellular activity. Therefore, for effective tissue regeneration, many researchers have been attempting to use decellularized extracellular matrix (dECM)-based materials [15,16,17,18]. To enhance the capability of bone regeneration, we produced dECM derived from bone tissue (bone dECM) and used it to cover the 3D scaffolds.

The present study aimed to evaluate the bone regeneration efficacy of customized 3D scaffolds covered with bone dECM for HTO. We conducted the analysis of scaffold characterization and in vitro tests, such as proliferation and differentiation. To confirm the capability of bone tissue regeneration, we performed in vivo experiments with beagles. For the customization of 3D scaffold implantation, we fabricated a customized guide instrument with a correction angle for an osteotomy zig and exploited it for scaffold implantation. Accordingly, microscopic computed tomography (micro-CT) analyses, quantitative data acquisition for bone regeneration, histological analyses, and radiologic evaluations were conducted.

## 2. Materials and Methods

**Figure 1:**
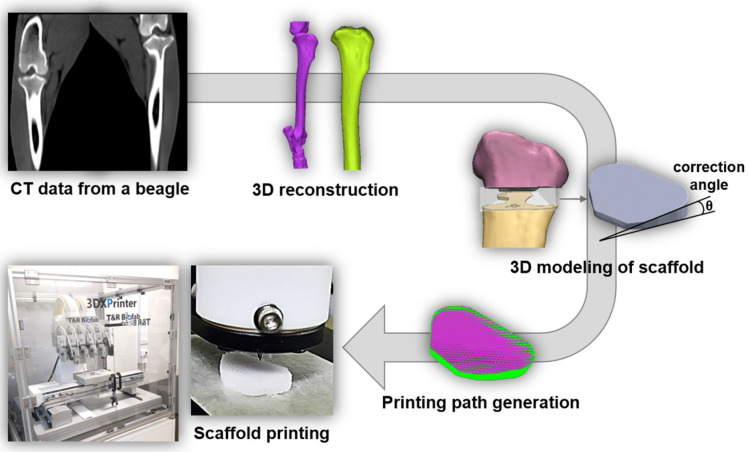
Three-dimensional (3D) printing process of scaffolds for high-tibial osteotomy (HTO).

For the preparation of bone-dECM, normal bone was collected from 8-month-old beagles at a slaughterhouse, and soft tissue, periosteum, bone marrow, and epiphyseal cartilage were removed from the collected bone. The bone was freeze-dried and pulverized using a commercial pulverizer (DSMP-370, DukSan Co., Ansan-si, Gyeonggi-do, Republic of Korea). The size did not exceed 4 mm and was stored at −80 °C. The freeze-dried and pulverized bone particles were decellularized to obtain the extracellular matrix. After that, the normal bone was demineralized in hydrochloric acid (HCl) solution for 12 h, and residual lipids were removed using a chloroform–methanol mixture (ratio 1:1). For cell removal, the demineralized bone powder was soaked in 0.05% trypsin and 0.02% ethylenediaminetetraacetic acid (EDTA) at 37 °C for 2 h. The samples were then rinsed with distilled water and 70% ethanol for more than 20 h. The resulting product, dECM, was lyophilized until needed. The bone dECM was digested in a pepsin solution (3 mg mL^−1^ pepsin in 0.05 N HCl) with continuous stirring for more than 72 h to obtain a final concentration of 20 mg of bone dECM per mL of pepsin solution. To fabricate a customized scaffold for the HTO, we reconstructed a 3D model from a beagle’s computed tomography (CT) image. Using this model, we designed and fabricated a customized scaffold for the HTO (Figure 1).

After fabricating scaffolds using polycaprolactone (PCL, PURASORB^®^ PC 12, Purac, Gorinchem, The Netherlands) and beta-tricalcium phosphate (β-TCP, 7758-87-4, Premier BioMaterials, Tipperary, Nenagh, Ireland) to form the bone, the PCL/β-TCP paste was loaded into a steel syringe and extruded through a steel nozzle using a 3D bioprinter (3DX printer, T&R Biofab Co., Ltd., Seoul, Republic of Korea) at a syringe temperature of 110 °C and a pneumatic pressure of 600 kPa. To prepare scaffolds covered with bone-dECM, the PCL/β-TCP scaffolds were immersed in the bone-dECM solution and freeze-dried for more than 48 h in an incubator at 37 °C. High-resolution scanning electron microscopy (SEM, Nova NanoSEM 450, Eindhoven, The Netherlands) analysis was performed to characterize the internal framework and chemical composition of the scaffold.

**Figure 2:**
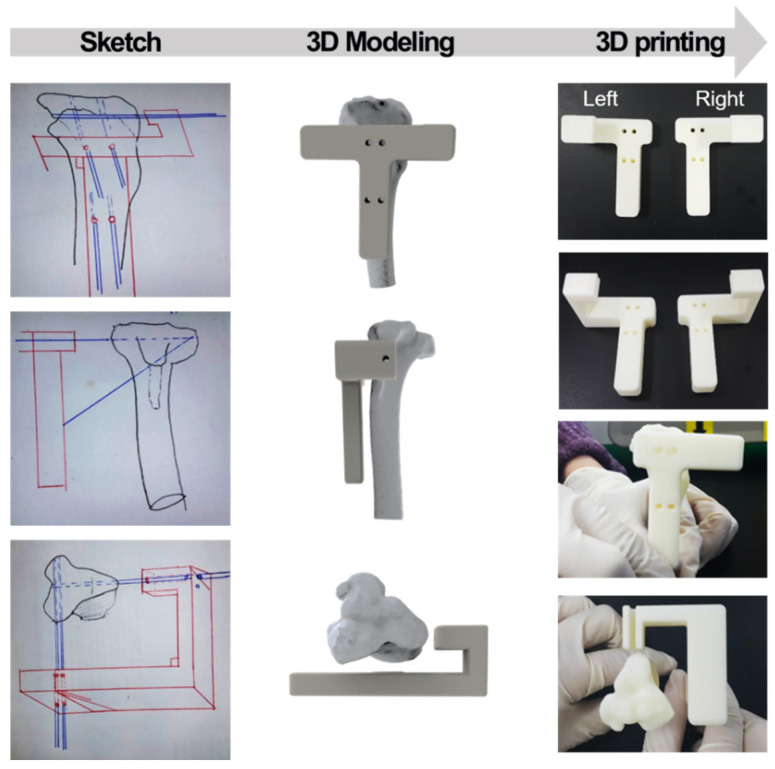
Sketch, 3D modeling, and fabrication of customized guide for osteotomy jigs.

Then, the HTO fracture jig guide was designed and prepared in a “T” shape with a width of 50 mm, a length of 43 mm, and a height of 63 mm. Four holes were drilled into the guide for rigid fixation to the bone. The guide was fabricated using a commercial 3D printer (uPrint SE, Stratasys, Eden Prairie, MN, USA). The osteotomy jig guide was designed for HTO with a lateral cortical hinge in the 3D plane [19]. This can prevent secondary changes in posterior tilt and distal tibial rotation. Three-dimensional simulation techniques were applied to model the synthetic augmentation material for the osteotomy. The acquired micro-CT image data were exported to Mimics (Materialise, Leuven, Belgium) to create a 3D model of the proximal tibia. This created a true anteroposterior (AP) view. According to the simulation, there was no secondary change in the posterior tibial inclination angle in the sagittal plane or rotational change in the axial plane (Figure 2). The aperture gap was set to about 10° in the coronal plane. After the HTO procedure, the angle between the upper and lower osteotomy plane X-ray images was measured for evaluation.

To validate the clinical efficacy of synthetic void fillers manufactured using 3D printing-based technology, 18 beagles were divided into groups according to whether or not they had lateral hinge fracture (LHF) during HTO [20]. LHF can occur as a complication of medial open wedge HTO during the formation of an open gap. It can lead to nonunion at the fracture site and loss of correction [21]. In the subsequent cell test, MC3T3-E1 (pre-osteoblast, #CRL-2593, ATCC, USA) cells were used. The cells were cultured in α-minimal essential medium (α-MEM, Gibco, USA) containing 10% fetal bovine serum (FBS, Gibco, Waltham, MA, USA) and 1% penicillin and streptomycin (Invitrogen, Waltham, MA, USA), and the medium was replaced every 2 days.

To determine the morphology of cell proliferation, the Cell Counting Kit-8 (CCK-8, Dojindo, Kumamoto, Japan) assay and the live/deadTM Viability/Cytotoxicity Kit (Life Technologies, Carlsbad, CA, USA) were used. The ratio of CCK-8 solution to culture medium was 1:10 and incubated with all scaffolds for 4 h in an incubator (around 37 °C, around 5% CO_2_). The cell number on the scaffolds was calculated using the OD value and standard value generated by MC3T3-E1 cells. The cell proliferation rate and cell morphology were checked at intervals of 1, 3, and 7 days. Live and dead cells were stained by mixing calcein AM, EthD-1, and phosphate-buffered saline (PBS) at a ratio of 1:2:500 and incubated with the samples for 30 min in an incubator (37 °C, 5% CO_2_). The samples were then washed three times with PBS. To determine osteogenic differentiation, cell-seeded scaffolds were cultured in osteogenic medium supplemented with α-MEM containing 20% FBS, 0.01 × 10^−3^ M dexamethasone, 0.2 × 10^−3^ M ascorbic acid, 10 × 10^−3^ M β-glycerol phosphate (Sigma-Aldrich, St. Louis, MO, USA), and 1% penicillin/streptomycin. To assay alkaline phosphatase (ALP) activity, cells cultured on scaffolds were lysed in radioimmunoprecipitation lysis buffer (Millipore, Burlington, MA, USA) at 4-, 7-, and 14-day intervals. The lysates were then incubated in a p-nitrophenyl phosphate (pNPP) liquid matrix system (Sigma-Aldrich, USA) at 37 °C for 30 min and read at 405 nm with a microplate reader.

The extracted data were utilized in two groups (PCL/β-TCP and PCL/β-TCP/bone-dECM) experiments. Beagles were anesthetized with a combination of zoletil (5 mg/kg) and xylazine (2 mg/kg) and maintained with isofurane (1.5–2.0%). For the beagle surgery, a medial OWHTO with a lateral cortical hinge was performed using a self-designed jig and 3D printer. The osteotomy was planned to start along the diaphyseal flare proximal to the tibial tuberosity. After the tarsal tendon was separated, the superficial medial collateral ligament was transected, and a guide wire was inserted toward the tip of the fibular head (safe zone) along the planned osteotomy plane [22]. The osteotomy was completed just anterior to the lateral cortex, and a scaffold was inserted into the osteotomy site. The correction angle was determined after scaffold insertion. The osteotomy was fixed with a fixed-angle plate with interlocking screws (TomoFixTM104, Synthes, Zuchwil, Switzerland), and the muscles and skin were sutured in a conventional manner. The micro-CT analysis was used to investigate the degree of mineralization at 8 and 16 weeks after beagle transplantation. Bone volume and density were also measured for quantitative analysis of bone regeneration, and bone regeneration capacity and mineral density (BMD) were determined using the Hounsfield Unit (HU) scale. For hematoxylin and eosin (H&E) staining, sacrificed samples were fixed in 4% paraformaldehyde for 7 days, decalcified in 10% ethylenediaminetetraacetic acid (EDTA) for 2 weeks, embedded in paraffin, and sectioned using a microtome (Leika RM2145 microtome, Wetzlar, Germany) to obtain sections of approximately 5 μm in thickness. For von Kossa staining, sacrificed samples were fixed in 4% formalin in PBS (pH = 7.4) for 7 days, dehydrated in alcohol, and embedded in methyl methacrylate. The surfaces of all sections were wet-polished with 35, 15, and 5 μm diamond pastes and ultrasonically cleaned to remove foreign matter. The sections were then ultraviolet-transmitted and treated with a 5% silver nitrate solution for 20 min, and 5% sodium triphosphate was added for von Kossa staining.

After the above procedure, gel permeation chromatography (GPC) analysis was performed to evaluate the degradation characteristics. The analysis was performed on an EcoSEC HLC-8320 GPC (Tosoh, Japan) equipped with a Shodex LF-804 column and Shodex KF-802.5 (7.8 × 3.00 mm^2^). The sample was dissolved in CHCl3 to adjust the concentration to 30 mg/mL and filtered using a 0.45 μm polytetrafluoroethylene filter. The molecular weight arrangement of the scaffold was confirmed using a refractive index detector at around 40 °C. The EcoSEC program was used for data analysis. Molecular weight was compared between initial implantation and 16 weeks later, and then the degradation rate of the implanted scaffolds was calculated. All data were expressed as the mean and standard deviation. Statistical significance was defined as * *p* < 0.05, ** *p* < 0.01, *** *p* < 0.005, and **** *p* < 0.001.

## 3. Results

### 3.1. Characterization of 3D Printed Scaffolds

**Figure 3:**
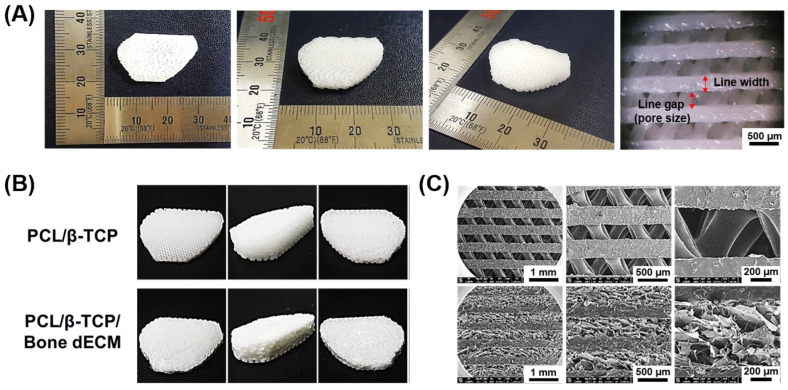
Characterization of scaffolds. (**A**) Optical images of the scaffolds with open wedge shape, (**B**) optical images, and (**C**) scanning electron microscopy (SEM) observation of two different scaffolds.

The line width and gap of the scaffolds with a 10° correction angle were 350 μm (Figure 3A). In this study, two different scaffolds were prepared to compare the effect of bone regeneration. PCL/β-TCP and PCL/β-TCP/bone dECM scaffolds were fabricated via 3D printing technology and a simple coating method with bone dECM (Figure 3B). On SEM imaging, the pore parts of the PCL/β-TCP scaffolds were cleaned, whereas the pore parts of the PCL/β-TCP/bone dECM scaffolds were filled with bone dECM (Figure 3C).

### 3.2. Characterization of Customized Guide Instrument

The osteotomy guide for HTO was a customized instrument for creating gaps with a certain size of the tibia part. For this purpose, the angles of the pin holes were set in advance to guide the saw direction into the tibia part. As shown in Figure 2, holes were designed to insert guide pins at a preset angle, and simulations were performed with a tibial model.

### 3.3. Cell Seeding Efficiency and Proliferation Results

**Figure 4:**
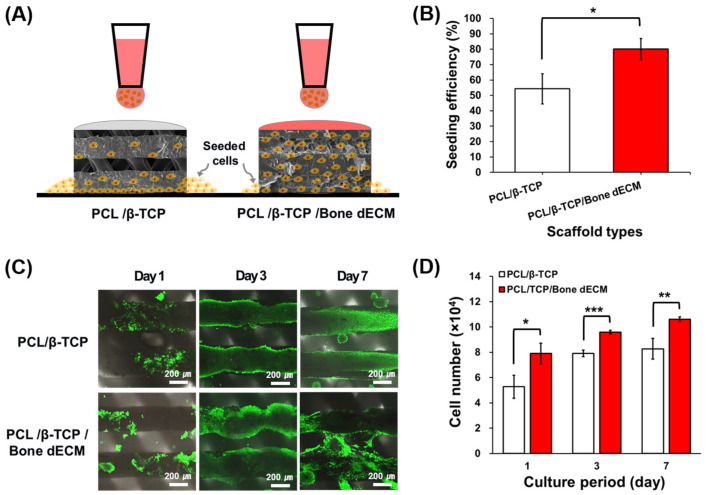
(**A**) Schematic of cell seeding on scaffolds; (**B**) cell seeding efficiency after seeding; (**C**) cell viability; and (**D**) cell proliferation outcomes for 7 days. The asterisks mean statistically significance.

Pre-osteoblasts (MC3T3-E1) were seeded on the PCL/β-TCP and PCL/β-TCP/bone dECM scaffolds (Figure 4A). Regarding cell seeding efficiency, PCL/β-TCP/bone dECM scaffolds yielded significantly higher cell seeding levels than those in the PCL/β-TCP scaffolds (*p* < 0.05, Figure 4B). At day 1, cells on the PCL/β-TCP and PCL/β-TCP/bone dECM scaffold yielded increased survival rates with >90% viability (Figure 4C). Live and dead staining and CCK-8 assays were performed on days 1, 3, and 7 for the analysis of cell viability and proliferation on scaffolds. As shown in Figure 4C, the cells in PCL/β-TCP scaffolds were only attached to the scaffold lines, whereas the cells in the PCL/β-TCP/bone dECM scaffold were attached to the scaffold line and bony dECM parts that filled the pore part. Throughout the entire culture period, PCL/β-TCP/bone dECM scaffolds exhibited excellent proliferation capacity compared with PCL/β-TCP scaffolds based on the initial cell seeding efficiency (Figure 4D). Based on the above results, the bone dECM is concluded to help with initial cell attachment and proliferation.

### 3.4. Cell Differentiation Results

**Figure 5:**
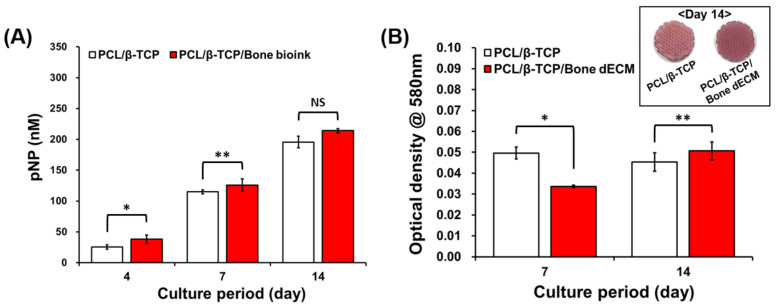
Cell differentiation results. (**A**) Alkaline phosphatase (ALP) activity analysis, and (**B**) quantitative analysis of alizarin red S staining. The asterisks mean statistically significance. NS: Not Significant.

To confirm the effect of bone dECM on osteogenic differentiation, ALP activity and alizarin red S staining were performed after the inducement of osteogenic differentiation with osteogenic media. ALP expression levels constitute an early osteogenic differentiation marker that statistically increased in PCL/β-TCP/bone dECM scaffolds compared with those related to PCL/β-TCP scaffolds at early time points (days 4 and 7). At day 14, the ALP expression level in the PCL/β-TCP scaffolds reached the same level as that of the PCL/β-TCP/bone dECM scaffolds (Figure 5A). Thus, bone dECM could promote early osteogenic differentiation. To confirm the mineralization, alizarin red S staining was performed on days 7 and 14. On day 7, PCL/β-TCP scaffolds that had direct contact between β-TCP and cells were more effective in osteogenic differentiation than PCL/β-TCP/bone dECM scaffolds. However, the PCL/β-TCP/bone dECM scaffold group yielded a higher level of mineralization at day 14 compared with that of the PCL/β-TCP scaffold group (Figure 5B). Moreover, the stained images showed the same result on day 14. Thus, the bone dECM did not only help the initial cell attachment and proliferation but also the osteogenic differentiation. It is thus concluded that bone dECM could provide an effective environment for bone tissue engineering.

### 3.5. In Vivo Results

**Figure 6:**
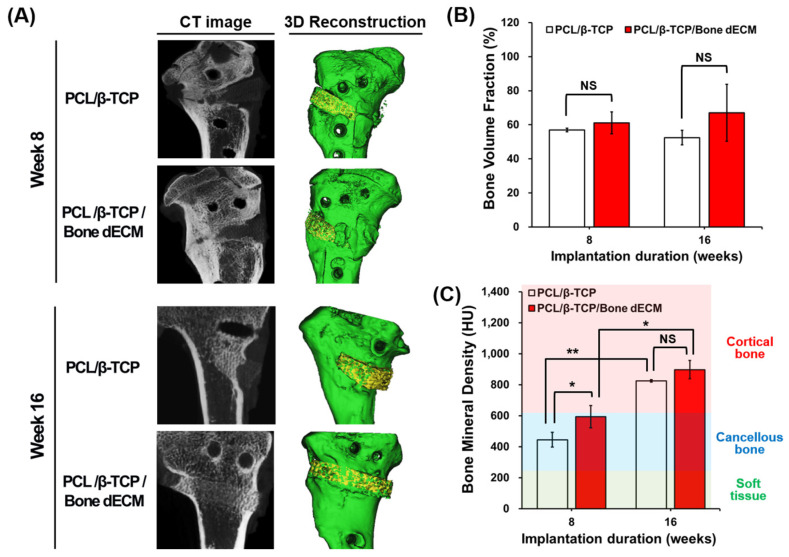
Bone regeneration results. (**A**) CT images and representative 3D reconstruction images; quantitative analysis of (**B**) bone volume fraction; and (**C**) bone mineral density at weeks 8 and 16 after implantation. The asterisks mean statistically significance. NS: Not Significant.

After harvesting the samples, micro-CT images were obtained to confirm the bone regeneration capacity of the scaffolds. Three-dimensional micro-CT models were reconstructed with images at weeks 8 and 16. In the 3D reconstructions, newly regenerated bone and native bone (including scaffold parts) were, respectively, presented with yellow and green colors (Figure 6A). For quantitative analyses of new bone formation at the defect site, the bone volume fraction (BV/TV, %) and bone mineral density (BMD) were calculated based on the HU values obtained from micro-CT data. Bone volume fractions increased as time progressed, even though these changes were not significantly different in the two scaffold groups throughout the entire period (Figure 6B). In the analyses of bone mineral density, soft tissue (~225 HU), cancellous bone (226–661 HU), and cortical bone (662–1988 HU) were separated based on the HU values [18]. At week 8, even though the HU values of PCL/β-TCP and PCL/β-TCP/bone dECM were comparable to those for cancellous bone, PCL/β-TCP/bone dECM scaffolds exhibited significantly higher HU values than PCL/β-TCP scaffolds (*p* < 0.05). At week 16, PCL/β-TCP and PCL/β-TCP/bone dECM had comparable densities to those of cortical bone. At week 16, PCL/β-TCP scaffolds had reached similar bone mineral densities compared with PCL/β-TCP/bone dECM scaffolds. This result suggested that bone dECM in scaffolds could help promote bone mineralization at early time points.

**Figure 7:**
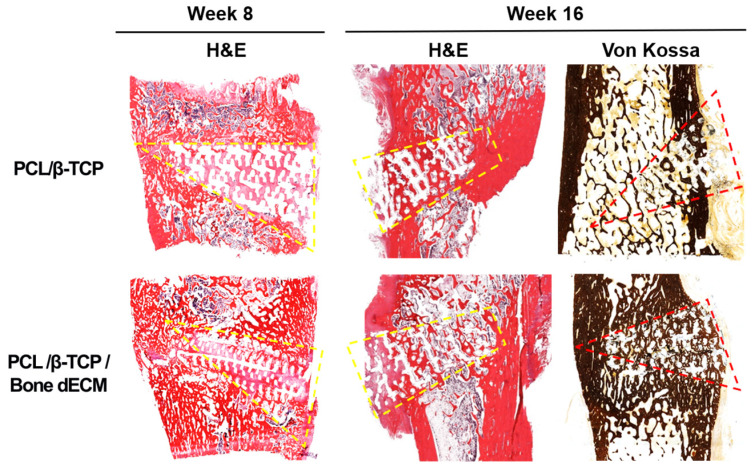
Histological analysis of implanted scaffolds based on hematoxylin and eosin and von Kossa staining at weeks 8 and 16. The discontinuous dotted lines (yellow and red) denote the implanted scaffolds.

To confirm the level of new bone formation in this animal test, histological analyses were performed (Figure 7). H&E and von Kossa staining were performed to confirm the new bone formation and mineralization. In the eighth week after implantation, H&E staining showed tissue penetration in the pores of the scaffolds, and partially new bone formation was documented in the rims of both implanted scaffolds. The PCL/β-TCP/bone dECM scaffold group yielded additional new bone formation than that documented in the PCL/β-TCP scaffold group in the area between the native bone and scaffolds. After 16 weeks, the area occupied by the scaffolds decreased, and new bone tissue was generated in the rest of the space. New bone tissue was confirmed and was mineralized by von Kossa staining. Specifically, the outcomes from the implanted PCL/β-TCP/bone dECM scaffolds confirmed that new bone tissue and bone marrow were combined. These results suggested that bone dECM could enhance the initial, new bone formation and mineralization.

### 3.6. Degradation Results

**Figure 8:**
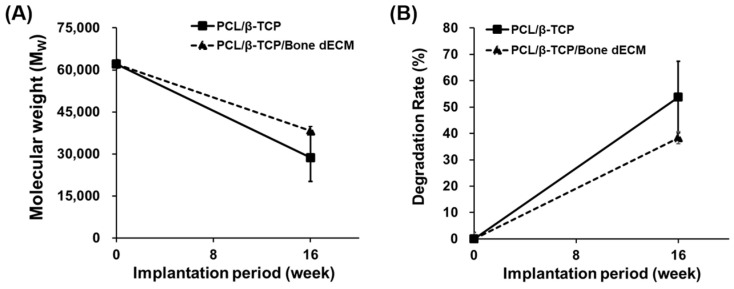
Degradation results. Variations in (**A**) molecular weight and (**B**) degradation rate of the implanted scaffolds for 16 weeks.

PCL and β-TCP are biodegradable polymers and exhibit natural degradation properties within the human body. To confirm the degree of degradation of the scaffolds during implantation, GPC analysis was performed at week 16 after implantation (Figure 8). PCL/β-TCP scaffolds were degraded by approximately 54% after 16 weeks. PCL/β-TCP/bone dECM scaffolds were degraded by approximately 38% after 16 weeks. Although there were differences in the extent of degradation, all the scaffolds showed that the in vivo degradation process was an ongoing process. Thus, scaffolds composed of PCL, β-TCP, and bone dECM had biodegradable properties and could be degraded in vivo and replaced with autologous tissues.

### 3.7. Radiologic Evaluations

The correction angle measured after osteotomy using synthetic void filler manufactured to attain an opening gap of 10° in coronal planes exhibited high accuracy with low errors (10.3 ± 0.9°). Additionally, even with LHFs, the correction angle was not significantly different (Table 1).

## 4. Discussion

Various studies have been published with scaffolds in the field of bone tissue engineering [12,13,14,15]. In particular, the bone regeneration effects of ceramic materials, such as HA and TCP, are well known because of their similar properties to bone tissues [13,14,18]. However, few studies show the synergistic effects of TCP and bone dECM incorporated in scaffolds. The present study was conducted to address two principal hypotheses. First, the construction of customized 3D scaffolds covered with bone dECM for HTO exhibited outstanding reparability for bone healing at the opening gap compared with the construct synthesized based on the isolation of the TCP from placed scaffolds at the knee joints of beagles. A closer analysis of the in vitro and in vivo test outcomes showed that scaffolds with bone dECM (PCL/β-TCP/bone dECM scaffolds) had superior structural capabilities, retained the surrounding cells at an early stage, and promoted cell attachment and proliferation. In addition, it was confirmed that differentiation was initially promoted by the bone-like microenvironment of bone dECM that contained various bone tissue-derived biomolecules, such as collagen and ECM proteins. However, as time progressed, it was confirmed that the bone regeneration effects of PCL/β-TCP scaffolds became similar to those of the PCL/β-TCP/bone dECM scaffolds. This indicates that β-TCP plays a major role in bone regeneration. Bone dECM produces good results (attachment, proliferation, and bone formation) in the early stages, but they cannot be maintained in the long term as it is degraded by enzymes in the body. In conclusion, as confirmed in this study, the synergistic effects of β-TCP and bone dECM were excellent. Specifically, in the case of scaffolds with relatively large volumes that are applicable to clinical trials, bone dECM contained in scaffolds could retain stem cells in the surrounding bone marrow. It is thought that they could promote bone healing.

For void fillers, autologous bone grafts are still the standard materials because they have osteogenic, osteoinductive, and osteoconductive effects [23]. Conversely, they also have some disadvantages, such as prolonged surgical times and additional donor site morbidities. Allogenous bone grafts that have no harmful effects on the donor site are a good alternative option to autologous bone grafts. However, they are associated with the potential risks of immune response or disease transmission [24]. Therefore, synthetic bone grafts, such as hydroxyapatite (HA) and TCP, are introduced for open wedge HTO. Previous studies have shown comparable results with allogenous bone grafts. Specifically, one of the advantages of synthetic grafts is that they can be initially grafted owing to their additional structural contribution if manufactured in wedge forms. However, several studies have demonstrated radiologically and histologically that synthetic grafts showed poor integration compared with other grafts [23,25]. The lack of graft absorption is still a concern after synthetic material grafting. From this point of view, scaffolds that use PCL have slow degradation properties and can provide the structural component for supporting the implant site during bone tissue regeneration. As confirmed by the degradation and histological results, molecular weight reduction of ~50% occurred after 16 weeks, but no structural collapse was observed in the histological results. This is a particularly important feature for bone regeneration that requires structural support. In animal experiments, the PCL/β-TCP scaffold also yielded excellent bone regeneration efficacy, but the PCL/β-TCP/bone dECM scaffold had a better initial bone regeneration efficacy. This means that bone regeneration was promoted based on (a) the ability of the scaffold to retain the bone marrow-derived stem cells at an early stage and (b) the bone tissue microenvironment of the bony dECM. PCL-based artificial scaffolds are known to have a degradation period of about 2 to 4 years in the human body. Because of such long degradation characteristics, there are few papers that show the entire period through animal experiments. In this study, the animal experiment period was set to 16 weeks to see the bone regeneration effect of PCL-TCP/bdECM scaffolds. Even when looking at the histological results of this animal experiment, a significant number of scaffolds remained. However, the molecular weight of the material was confirmed to have decreased by about 50% through the GPC results. This confirmed that the material was degraded by hydrolysis.

Second, this 3D printing-based platform technology can be effectively exploited to attain the desired correction angle with preoperative simulations. Synthetic void filler manufactured with 3D printing-based platform technology according to preoperative simulations was exploited for scaffold implantation with the use of a fabricated customized guide instrument with a correction angle for an osteotomy zig. Following postoperative radiologic evaluations in 18 beagles, the correction angles yielded high accuracy and reliability regardless of the absence or presence of LHF at the HTO procedure. An LHF may occur as a complication of medial open-wedge HTO during the formation of the opening gap, which causes instability at the osteotomy site and may lead to delayed union or nonunion. Correspondingly, it could thus result in correction losses after the medial open-wedge HTO [21]. Therefore, our technologies showed the new clinical availability of synthetic void filler manufactured by 3D printing-based platform technology using preoperative simulation. The filling methods used for successful gap healing in the formed gap created during open-wedge HTO—which is a surgical option for medial compartmental osteoarthritis or osteonecrosis with varus deformity of the knee joint [26,27]—would lead to a wide clinical applicability in the orthopedic field, including nonunion, osteomyelitis, and malunion correction.

Given that 3D printing technologies enable free-form fabrications, these technologies are extensively used in the fabrication of porous structures that are advantageous for inducing tissue regeneration [12,28,29,30]. Particularly, extrusion-based 3D printing systems have been applied in the fields of various tissue regeneration and regenerative medicine because of their ability to use a variety of biopolymers, such as PCL [12,29,30]. For bone tissue regeneration, ceramics, such as hydroxyapatite (HA) and TCPs, have been considered potential candidates for effective bone reconstruction [29,30,31,32]. Furthermore, some studies have conducted bone-tissue-derived biomaterials that consist of ECM components of bone tissue for enhancing bone tissue regeneration [15,16,17].

For these reasons, we fabricated the customized scaffolds using PCL, β-TCP, and bone dECM. These scaffolds were customized structures designed and printed with the desired correction angle for open-wedge HTO. The scaffolds were composed of (a) PCL that constitutes a supporting part and (b) β-TCP and bone dECM that can promote bone tissue regeneration. The β-TCP in the scaffolds play important roles in bone regeneration owing to their composition but also have roles in the promotion of the degradation rate of PCL by expanding its surface area given that its degradation rate (approximately 3–6 months) is faster than that (about over 12 months) of PCL [30]. Additionally, bone dECM consists of bone tissue in the microenvironment and is thus effective for bone regeneration. This is a key feature that can overcome the disadvantage that the cells are attached only to the surface of the porous scaffolds and the fact that it is difficult to retain many cells within the scaffolds. Nevertheless, these cells possess tremendous potential when seeded in scaffolds for effective bone tissue regeneration.

## 5. Conclusions

The customized 3D scaffold with PCL/β-TCP/bone dECM showed excellent outcomes regarding cell seeding efficiency, proliferation capacity, early osteogenic differentiation, and bone mineralization. Given that the scaffolds provide the proper environment that promotes bone tissue regeneration, their capacities to achieve their integrations with native tissue in vivo were excellent. It is expected to solve the disadvantages related to bone union in MOWHTO and to replace autografts in the future.

## Figures and Tables

**Table 1:** Comparison of post-high-tibial osteotomy (HTO) correction angle according to the presence of hinge fractures.

Grouping According to LHF	Nonlateral Group (N = 10)	LHF Group (N = 8)	*p* Value
Correction angle (°)	10.6 ± 0.7	9.8 ± 1.0	0.087

LHF: Lateral cortical hinge fracture.

## Data Availability

Data are contained within the article.
